# Advances in Portable and Wearable Acoustic Sensing Devices for Human Health Monitoring

**DOI:** 10.3390/s24165354

**Published:** 2024-08-19

**Authors:** Fanhao Kong, Yang Zou, Zhou Li, Yulin Deng

**Affiliations:** 1 School of Medical Technology, Beijing Institute of Technology, Beijing 100081, China; kongfanhao@bit.edu.cn; 2 Beijing Institute of Nanoenergy and Nanosystems, Chinese Academy of Sciences, Beijing 101400, China

**Keywords:** acoustic sensors, portable and wearable, health monitoring, clinical diagnosis

## Abstract

The practice of auscultation, interpreting body sounds to assess organ health, has greatly benefited from technological advancements in sensing and electronics. The advent of portable and wearable acoustic sensing devices marks a significant milestone in telemedicine, home health, and clinical diagnostics. This review summarises the contemporary advancements in acoustic sensing devices, categorized based on varied sensing principles, including capacitive, piezoelectric, and triboelectric mechanisms. Some representative acoustic sensing devices are introduced from the perspective of portability and wearability. Additionally, the characteristics of sound signals from different human organs and practical applications of acoustic sensing devices are exemplified. Challenges and prospective trends in portable and wearable acoustic sensors are also discussed, providing insights into future research directions.

## 1. Introduction

The scientific community has long been fascinated with the identification and clinical application of human physiological acoustic signals. Over the past several decades, modern medicine’s continuous evolution and integration with sensing technologies have prompted the emergence of an assortment of portable and wearable acoustic sensing devices. These devices are designed to efficiently and conveniently detect a range of physiological acoustic signals, providing critical data for clinical diagnosis, which play a vital role in areas such as telemedicine, home health care, and early screening. Given their non-invasive, ease of use, high efficiency, and ability to provide continuous monitoring, these acoustic sensing devices are anticipated to transform human health monitoring methods radically.

The range of principles behind acoustic sensing has spawned a variety of sensors that can capture acoustic signals and transform them into electrical outputs. These sensors provide a crucial means for direct acoustic signal detection. In this review, we begin by summarizing the fundamental principles of these acoustic sensors, with a particular focus on capacitive, piezoelectric, and triboelectric sensors. Different acoustic sensing principles provide multiple choices for human acoustic detection. However, these acoustic sensors need further integration into corresponding detection devices for practical application. Additionally, as these devices are intended for human body detection, they must ensure a high-quality user experience despite being non-invasive. Here, we categorize the evolving acoustic sensing devices into two types: portable and wearable acoustic sensing devices. Portable acoustic sensing devices emphasize ease of use and the ability to be used anytime and anywhere, including stethoscopes and electronic stethoscopes. On the other hand, wearable acoustic sensing devices prioritize long-term continuous monitoring and comfort, including skin patches, electronic fabrics, and bionic microelectromechanical systems (MEMS). This article will introduce some of the representative advancements in both types of acoustic sensing devices.

A recent review by Jeng-Hun Lee et al. describes the progress of soft acoustic and vibration sensors over the past decade, emphasizing machine learning integration into signal processing to improve the interpretation and accuracy of data obtained from these sensors [1]. Zhiwei Lin et al. also discuss the recent advances and applications of flexible and wearable acoustic sensors, highlighting key fabrication methods for transitioning acoustic sensors from traditional rigid forms to innovative, flexible wearable devices [2]. In this review, we primarily focus on the application of various acoustic sensing devices in health monitoring by detecting different acoustic signals emitted by the human body, which can reflect the physiological health status of an individual. The characteristics and existing medical understanding of some important human body acoustic signals commonly examined in clinical practice are further summarized. Meanwhile, some typical cases of practical applications of acoustic sensing devices designed for detecting cardiac, pulmonary, and gastrointestinal sounds are also provided. Last, we discuss the challenges currently faced by acoustic sensing devices in healthcare and offer insights into their potential future directions.

## 2. Acoustic Sensors

Acoustic sensors are devices that convert acoustic signals into electrical signals, serving as an essential instrument across diverse scientific and technological disciplines. Acoustic sensors have been widely used in many fields, such as environmental monitoring, information security, human–machine interaction, clinical diagnosis, and health monitoring [3,4]. In this paper, we categorize acoustic sensors based on the principle of acoustic sensing. We divide them into capacitive sensors, piezoelectric sensors, and triboelectric sensors. In practical applications for sound detection, these sensors are commonly utilized in the form of devices such as microphones or accelerometers. When sound waves reach the microphone, they cause a vibrating component (such as a diaphragm or capacitor plate) to move, leading to a change in electrical charge on the electrodes of the vibrating component, allowing the microphone to output a corresponding electrical signal. An accelerometer is a sensing device that measures acceleration forces. This occurs by measuring the displacement or force caused by inertial acceleration and converting the displacement acting on a sensitive element into an electrical signal for output [5]. The subsequent sections provide a detailed introduction to acoustic sensors, delineating their varied operational principles and practical cases.

### 2.1. Capacitive Sensor

**Figure 1:**
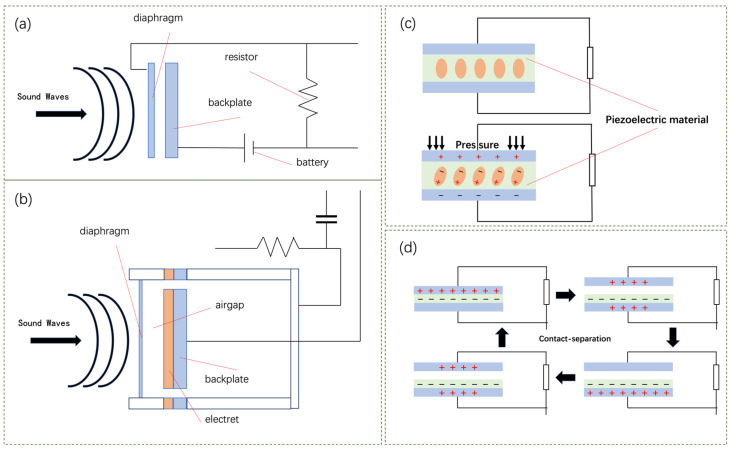
Principles of various types of acoustic sensors. (**a**) Schematic diagram of capacitive microphone. (**b**) Schematic diagram of the electret microphone. (**c**) Schematic diagram of piezoelectric sensor. (**d**) Schematic diagram of triboelectric sensor.

Capacitive acoustic sensors are transducers that operate on the principle of capacitance change. They are among the most widely used and high-performing acoustic sensors available today. Condenser microphones, designed to work within the human auditory range of 20 to 20,000 Hz, are a type of acoustic sensor at the core of which is a capacitor formed by a flexible diaphragm and a relatively fixed metalized backplate (Figure 1a). The diaphragm is typically made from a metalized polyester film, which is extremely thin and possesses a certain degree of elasticity, allowing it to respond sensitively to pressure changes induced by sound waves. The backplate is precision-engineered, with a pattern of perforations on its surface to ensure even air pressure distribution and unobstructed transmission of sound waves. The diaphragm and backplate form a pair of parallel plates with an air-filled gap as the dielectric medium. A bias voltage, also known as a polarization voltage, is usually applied across the plates to maintain a constant charge. When sound waves cause the diaphragm to vibrate, the changing distance between the diaphragm and the backplate results in a change in capacitance. Since the charge on the capacitor’s plates remains constant, any change in capacitance leads to a change in voltage. This voltage change is transmitted via wires to the input circuit (usually an impedance-matching preamplifier circuit), and after signal conditioning, it can be further amplified, processed, and recorded.

Condenser microphones boast impressive features such as high sensitivity, wide dynamic range, broad and flat frequency response, superior transient response and stability, extremely low sensitivity to mechanical vibration, and excellent sound quality. However, due to their complex manufacturing process and high cost, condenser microphones are extensively used in high-fidelity recording situations such as television, broadcasting, film, and theater or for precise acoustic measurements in scientific research [6,7]. Furthermore, due to their highly stable sensitivity and the capability of absolute calibration, they can be precisely calibrated in voltage terms and used as an acoustic reference standard.

Electret microphones are a specific type of condenser microphone that incorporates a pre-polarized electret material for the diaphragm or backplate (Figure 1b). Electret materials are dielectrics that can retain an electric polarization state for an extended period, such as polytetrafluoroethylene (PTFE), which carries a permanent charge after being polarized by a strong electric field. As a result, electret microphones do not require a bias voltage across the plates during operation, and changes in capacitance can be directly converted to changes in voltage signal, simplifying the circuitry and reducing power consumption. Electret microphones are known for their cost-effectiveness, compactness, and high sensitivity to sound, making them particularly popular for portable and remote recording applications. However, electret microphones typically offer a relatively narrow dynamic range and may be susceptible to environmental conditions such as humidity and temperature. Prolonged exposure to harsh environments could potentially lead to a decline in performance.

To address the limitations of conventional condenser microphones in detecting low-frequency sounds, Kumjae Shin et al. proposed a solution of an electret gate on a field effect transistor (ElGoFET) microphone. They introduced a displacement-based transconductance system, which consists of a combination of a field effect transistor (FET) and an electret. An electric field from the electret substrate modulates the channels of the FET embedded in the diaphragm, and the FET mounted on the diaphragm vibrates because of the external acoustic pressure, thereby changing the distance between the FET channels and the electret, and the conductivity of the channels is modulated by the resulting change in electric field. Thus, diaphragm displacement can be directly detected using a transduction mechanism consisting of an integrated FET and an electret. This transduction mechanism is generated by the electret gate on a field effect transistor (ElGoFET) [8].

Capacitive accelerometers can also serve in acoustic detection. However, they are generally better suited for measuring structural vibrations induced by sound waves rather than directly measuring them. These devices hold unique advantages in the monitoring of building acoustics or the analysis of machinery operation noises. A capacitive accelerometer measures acceleration using a sensitive micro-mass block that forms a capacitor with one or more fixed electrodes [9]. The mass block is connected to the body of the accelerometer through delicate elastic structures, such as cantilever beams or micro-springs [10,11]. In a state of rest or uniform motion, where no acceleration is present, the mass block maintains a constant position relative to the fixed electrodes, keeping the capacitance unchanged. When the accelerometer is subjected to acceleration (such as vibration), the mass block displaces relative to the main body due to inertia, altering the distance to the fixed electrodes and thus changing the capacitance. Electronic circuits detect these changes in capacitance and convert them into electrical signals, which are then amplified and processed to realize a measurement of acceleration.

Due to their ability to detect extremely subtle vibrations, wide bandwidth response, and high sensitivity, capacitive accelerometers are employed to monitor vibrations induced by sound waves in solid media, such as mechanical equipment, building structures, or human organs. In such applications, accelerometers can assist in identifying structural resonance or analyzing acoustic properties. Nevertheless, to achieve precise vibration measurements, accelerometers usually need to be firmly mounted on the surface of the object being measured, which could limit the scenarios of their application. Recent research on capacitive accelerometers has shown a shift towards medical and healthcare applications. Pranav Gupta and colleagues have described a high-bandwidth, low-noise, unidirectional capacitive accelerometer featuring a deformable frame and a vibration damper—all enclosed within a hermetically sealed package. This design enables the accelerometer to achieve bandwidths exceeding 14 kHz, which allows it to capture human speech and heart sounds with high fidelity while minimizing the intrusion of ambient noise [12]. Such advancements underscore the potential of capacitive acoustic sensors in precise health monitoring and diagnostic applications, where capturing the subtle nuances of biological sounds is crucial.

### 2.2. Piezoelectric Sensor

Piezoelectric sensors are devices that utilize the piezoelectric effect to measure various dynamic forces, mechanical impacts, and vibrations, finding widespread applications in acoustics, mechanics, medicine, and other fields. The piezoelectric effect occurs in certain dielectric materials when an external force is applied along a specific direction, causing deformation and displacing the positive and negative charge centers within the material, resulting in polarization [13]. This produces opposite charges on two surfaces of the material (Figure 1c). When the force is removed, the material reverts to an uncharged state. If the direction of the force changes, the polarity of the charges also reverses. Materials that exhibit this phenomenon are referred to as piezoelectric materials.

By exploiting this property, a piezoelectric sensor can be created by depositing electrodes on the symmetrical surfaces of piezoelectric materials, thus forming a device that converts mechanical deformation into an electrical signal [14]. Due to their very high internal resistance, these sensors typically require a preamplifier circuit with high input impedance to convert the high-impedance output of the sensor to a low-impedance output and amplify the weak electrical signals. Common piezoelectric materials include piezoelectric crystals, piezoelectric ceramics, piezoelectric semiconductors, and piezoelectric polymers. In practical applications, the choice of piezoelectric material must be tailored to the specific requirements of the application to design and construct the appropriate sensor. Piezoelectric polymer films, which can be produced on a large scale and formed into substantial areas, have a unique advantage in matching the acoustic impedance of air, making them promising new electroacoustic materials with significant development potential.

Piezoelectric sensors are extensively used in acoustic sensing because of their high sensitivity and broad frequency response. These sensors serve as critical tools for detecting and measuring sound waves in areas such as sonar detection, structural health monitoring, and medical diagnostics such as ultrasonography. Acoustic waves induce stress on the surface of piezoelectric sensors, causing deformation proportional to the wave’s intensity and frequency, ensuring accurate acoustic signal transduction. The resultant deformation generates charges on the material’s surface induced by electrodes for measurement. Thus, sound waves are converted into electrical signals for subsequent circuit processing and analysis. The shape, size, and structural configuration of a piezoelectric sensor can influence its sensitivity and frequency response, with different applications requiring the sensor to function within specific frequency ranges. Therefore, the sensor’s resonant frequency can be adjusted by changing the thickness of the piezoelectric material or by incorporating specific structures. Additionally, the shape of the sensor can be optimized based on the propagation characteristics of sound waves to enhance directionality or focusing capability.

As described, piezoelectric acoustic sensors are highly delicate and adjustable devices capable of precisely converting sound waves into electrical signals. Furthermore, they can be tailored and optimized to meet specific application requirements. This adaptability renders piezoelectric acoustic sensors incredibly versatile, finding extensive applications across a multitude of scientific and industrial fields, demonstrating the unique value of piezoelectric technology in enhancing the capabilities of modern sensing and diagnostic tools.

Piezoelectric acoustic sensors based on micro-electro-mechanical systems (MEMS) technology represent a significant avenue of research for next-generation acoustic sensing due to their small size, high level of integration, and low power consumption. Compared with bulk piezoelectric substrates such as quartz or lithium niobate (LiNbO3), piezoelectric thin films such as zinc oxide (ZnO), aluminum nitride (AlN), and lead zirconate titanate (PZT) offer greater design flexibility, which is more conducive to integration into complex MEMS systems [15]. To fabricate these piezoelectric thin films, manufacturing processes such as sputtering or sol-gel methods are typically employed, enabling the deposition of piezoelectric materials in ultra-thin layers on traditional substrates such as glass or silicon. These methods are utilized to construct high-frequency surface acoustic wave (SAW) devices and thin-film bulk acoustic resonators (TFBARs), critical components in various high-precision acoustic sensing applications. E.S. Kim. et al. mentioned a ZnO piezoelectric microphone with a size of 2 mm square [16], which was made using a micromachining process combined with a ZnO thin film layer as a piezoelectric transducer. The microphone consists of a ZnO layer and multiple ring electrodes deposited on a micromachined diaphragm made of LPCVD silicon nitride. M. D. Williams et al. present an AlN MEMS piezoelectric microphone [17], which achieves piezoelectric conversion through an integrated aluminum nitride layer in a thin-film composite diaphragm. The fabrication process includes surface and integral micromachining, including etching of shallow cavities, wafer thinning, deposition and patterning of layers, and etching of sacrificial material to release the diaphragm. Yi-Ping Zhu et al. present the design, fabrication and test results of a novel in-plane polarized PZT thin film micro acoustic device [18] for ultrasonic applications. The device utilizes a Pt/PZT/TiO_2_/SiO_2_ structure and can be used as a microphone and micro-speaker.

The piezoelectric accelerometer is a specific type of piezoelectric sensor that typically couples a piezoelectric element with a mass block to detect inertial force, which is known for its wide range of applicability, simple structure, and stable performance [19]. A. L. Gesing et al. explore the design of MEMS piezoelectric accelerometers coupled to the middle ear, which are intended for use as implantable sensors for hearing devices. Different piezoelectric accelerometer designs were optimized using finite element modeling to meet criteria such as compactness, low internal noise, low power consumption, and large bandwidth. Upon evaluation of the prototype, the performance of the MEMS piezoelectric accelerometer demonstrated strong concordance with the models, exhibiting close alignment within the frequency range of 100 Hz to 24 kHz [20].

Piezoelectric acoustic sensors utilizing novel materials demonstrate increased potential for human health monitoring applications. Md Osman Goni Nayeem et al. developed an all-nanofiber mechano-acoustic piezoelectric sensor for long-term continuous cardiac monitoring. The nanofiber electrode layer enables the sensor to vibrate more in response to acoustic waves, thus achieving high sensitivity. The piezoelectric sensor is not only lightweight and breathable but also exhibits excellent sensitivity of 10,050.6 mV Pa^−1^ and a wide dynamic range [21].

### 2.3. Triboelectric Sensor

Triboelectric sensors are a type of self-powered sensor based on the triboelectric nanogenerator (TENG), which can directly convert mechanical stimuli such as friction, vibration, and deformation into electrical signals without the need for an external power source. TENG-based triboelectric sensors exhibit high sensitivity, easily capturing subtle mechanical changes and offering a broad operating frequency range and environmental adaptability, along with advantages such as simple structure, a broad selection of fabrication materials, cost-effectiveness, flexibility, portability, and high customizability. They hold significant application potential in fields such as the Internet of Things (IoT), health monitoring, human–machine interaction, environmental surveillance, and smart transportation [22]. Since Professor Zhonglin Wang first introduced the TENG concept in 2012, these devices have garnered considerable attention for energy harvesting and self-powered sensing [23]. Over more than a decade, TENG research has evolved from initial proof-of-concept and fundamental theory to performance optimization, exploration of new materials, and multifunctional integrated devices. Researchers have significantly enhanced TENGs’ energy conversion efficiency and power density by designing novel structures, utilizing diverse tribo-materials, and integrating various effects such as photoelectric and piezoelectric. Moreover, by meticulously controlling the microstructure of the triboelectric layer interface or applying surface modifications, researchers can tune the output characteristics of TENGs to suit different application scenarios better.

The basic structure of TENG is simple, typically consisting of two materials with differing electron affinities and back electrodes. The working principle of TENGs is based on the triboelectric effect, involving contact electrification and electrostatic induction, and operates in four basic steps [24]: (1) Contact electrification: When two different materials come into contact, their varying electron affinities lead to charge transfer, accumulating positive charges on one material’s surface and an equal number of negative charges on the other. (2) Separation: As the charged materials separate, a potential difference is induced across the electrodes due to the charge disparity, causing electrons to flow through an external circuit from one electrode to the other to balance the charges. (3) Electron flow: The flow of electrons in the external circuit constitutes a current, the direction and intensity of which depend on the charge variation on the material surfaces and the separation velocity. (4) Recontact: When the two materials come into contact again, the charges redistribute in an attempt to return to the initial equilibrium state. The redistribution of charges prompts electrons to flow in the reverse direction, thus generating a reverse current. This cycle repeats continuously; with each contact and separation of the materials, electrons flow back and forth in the external circuit, thereby generating a sustained alternating current (AC) pulse signal [1]. The TENG design can take various forms, such as vertical contact-separation mode, lateral sliding mode, single-electrode mode, and freestanding mode, each with its unique operating mechanism and application scenarios.

Triboelectric acoustic sensors are capable of directly converting the mechanical vibrations of sound waves into electrical signals. They are employed to detect the presence, frequency, amplitude, and other attributes of sound waves, offering numerous advantages such as self-powering, high sensitivity, a broad frequency response, and a lightweight, even flexible design. Based on the unique capacitive model of triboelectric technology, Huake Yang et al. prepared a novel self-powered triboelectric acoustic sensor (T-MIC). The TENG-based acoustic sensor offers higher performance and cost advantages over conventional acoustic sensors by sensing sound waves over the full frequency range of human hearing (20 to 20,000 Hz), with a high sensitivity (21.50 times) and frequency resolution (1 Hz accuracy) [25]. Yingzhe Li et al. also proposed a self-powered acoustic sensor utilizing triboelectric nanogenerator technology. The sensor consists of an adjustable pitch structure and a sound-driven TENG, which exhibits excellent electrical output characteristics because of its porous electrode structure, ultrathin vibrating membrane, and high-quality triboelectric material. The transducer, whose electrical output frequency is closely related to the applied sound wave and whose direction-dependent mode is highly symmetric, produces a maximum output voltage of 6.28 V, an acoustic frequency of 350 Hz, and a sound pressure of 110 dB [26].

### 2.4. Comparison and Summary of Different Acoustic Sensors

Table 1 provides a summary and comparison of the various acoustic sensors mentioned above, highlighting the principles, characteristics, limitations, and applicability of each type of sensor.

Condenser microphones and electret microphones are the most common commercially available acoustic sensors today, both utilizing the principle of capacitance change to convert sound waves into electrical signals. Condenser microphones typically have high sensitivity, wide frequency response, broad dynamic range, and low sensitivity to mechanical vibration. Their excellent sound quality makes them suitable for high-fidelity recording and precision acoustic measurements. However, condenser microphones require an external power source, such as phantom power, to support their superior sound quality and dynamic range when in use, leading to a more complex and expensive manufacturing process. In contrast, electret microphones utilize a pre-polarized electret material, eliminating the need for external power thereby making them more compact and cost-effective. These microphones are commonly integrated into portable and everyday audio devices such as mobile phones and headphones. Nevertheless, they exhibit a narrower dynamic range and are more susceptible to environmental conditions such as humidity and temperature variations.

Capacitive accelerometers and piezoelectric accelerometers are both used to measure the acceleration of the object and are highly sensitive to impact and vibration. When used for acoustic sensing, they are usually more suitable for measuring structural vibrations caused by sound waves, which can help identify structural resonances and analyze acoustic characteristics. These devices have unique advantages in monitoring the vibrations caused by sound waves in solid mediums, such as mechanical equipment, building structures, or human organs, and are widely used in machinery fault detection, building acoustics monitoring, and health monitoring. However, to achieve precise vibration measurement, accelerometers typically need to be firmly mounted on the surface of the object being measured, which may limit their application scenarios. Capacitive accelerometers offer high sensitivity, wide bandwidth, and the ability to measure static and dynamic signals but require an external power supply and are susceptible to shock and vibration. On the other hand, piezoelectric accelerometers have a good high-frequency response, simple structure, and good stability and do not require an external power supply. However, the low-frequency response is poor, and they can not measure static signals.

Piezoelectric and triboelectric acoustic sensors both convert mechanical energy into electrical signals, yet they utilize different mechanisms and materials to achieve this. Both types of sensors are known for their high sensitivity and the lack of an external power supply, but because of the high output impedance, matching circuits are usually required. Piezoelectric acoustic sensors utilize the piezoelectric effect, where the deformation of the piezoelectric material caused by sound waves generates electrical charges to achieve sound detection. With the characteristics of simple structure, fast response speed, and resistance to electromagnetic interference, they are suitable for use as musical instrument pickups or for structural health monitoring and also play an important role in ultrasound detection and medical diagnosis. However, they have relatively poor low-frequency response and higher costs. Triboelectric acoustic sensors utilize the triboelectric effect and electrostatic induction to generate charges by contact and separation of materials with different electron affinities caused by sound waves. These sensors have characteristics of wide frequency response, simple structure, a wide range of material choices, and high cost-effectiveness. In recent years, as a novel type of acoustic sensor, they have great potential for applications in the Internet of Things, human health monitoring, environmental monitoring, and other fields. Despite their promising potential, triboelectric acoustic sensors face challenges related to improving their stability and consistency under various environmental conditions, and the durability of the material is also an issue to be considered.

## 3. Portable and Wearable Acoustic Sensing Devices

Traditional acoustic sensors are often limited by their rigid and bulky shape, which restricts their potential applications. The recent trend towards miniaturization and smart design has facilitated the development of portable and wearable acoustic devices that conform to soft, curved, and deformable surfaces, resulting in a more comfortable user experience and a friendlier user interface [2]. Attributable to their convenience and high efficiency, portable and wearable acoustic sensors have secured a critical role across diverse domains such as health monitoring, disease diagnosis, telemedicine, and human–machine interaction. These innovations are reshaping the landscape of personal and healthcare devices, providing seamless integration into everyday life and enabling continuous, real-time health monitoring. Here, we will summarize and introduce the relevant advances of acoustic sensing devices, mainly from the perspectives of portable and wearable devices for human health monitoring.

### 3.1. Portable Devices

Among portable acoustic sensors used for human health monitoring, the stethoscope is undoubtedly one of the most successful cases in practice. Auscultation has long been a cornerstone of medical practice, and the stethoscope, introduced in the 1820s, has been an indispensable diagnostic tool for over two centuries, offering real-time and information-rich insights for medical practitioners. The evolution of the stethoscope has led to the incorporation of electronic and digital technologies, enhancing sound quality, reducing background noise, and enabling the integration of computer-aided systems for more sophisticated analytical capabilities. These advancements have significantly augmented the utility of the stethoscope in clinical settings, paving the way for more precise and effective patient assessments.

#### 3.1.1. Stethoscope

The practice of chest auscultation remains a crucial element in diagnosing respiratory and heart conditions. Furthermore, auscultation is also employed in the clinical assessment of peripheral vessels, gastrointestinal tract, and other internal organs ailments [27]. Due to the simplicity and efficiency of the stethoscope, it plays a significant role as a portable clinical diagnostic tool, especially in the conduct of physical examinations. A stethoscope can quickly provide doctors with valuable insights into the patient’s health condition by listening to and assessing the internal sounds of a patient’s body.

A stethoscope comprises three parts: the chest piece, the leather tube, and the earplugs. The chest piece is the most critical component of a stethoscope, typically consisting of a flat diaphragm and a hollow bell. The diaphragm is used for detecting high-frequency sounds, while the bell is better suited for capturing low-frequency sounds. Some modern stethoscopes have a dual-sided chest piece that allows the user to select between the diaphragm or the bell by rotating it. The leather tube is the part that carries the sound and connects the chest piece to the earbuds. The earplugs are the part of the stethoscope that inserts into the ears, which can be a soft-sealing or hard earplug. The stethoscope works on acoustic principles, capturing sound waves through a chest piece placed on the patient. During auscultation, the diaphragm vibrates in response to the surface of the body, and the vibrations of the diaphragm transmit the sound waves through a hollow tube to the ears of healthcare professionals, who can make clinically sound judgments about the patient based on the loudness and subtle characteristics of these sound waves.

In 1816, the French physician René-Théophile-Hyacinthe Laënnec invented the first stethoscope while examining a young woman suspected of having a heart condition [28]. In 1851, Dr. Georges-Philippe Carman invented the binaural stethoscope, which consisted of braided tubes, a wooden chest piece, ivory earplugs, and wide rubber bands for fastening. In 1961, Dr. David Littman used a simple model of a stethoscope, modeled in the shape of a “Y”, to form a double-headed chest piece [29], which is probably the most widely used stethoscope prototype in modern times [30,31]. In the ensuing period, binaural stethoscopes gradually gained popularity and became essential clinical diagnostic tools worldwide.

However, because high-tech equipment such as ultrasound machines may be limited or unavailable in resource-limited settings, stethoscopes are widely available and cost-effective. More than that, in many clinical scenarios, stethoscopes provide a quick and efficient way to gather important information. Stethoscopes allow for a quick initial assessment of a wide range of conditions (especially those related to the heart and lungs) during an emergency or routine checkup, which is critical when it comes to obtaining an immediate understanding of a patient’s health status [32].

With the development of modern medicine and the advancement of technology, a host of advanced clinical diagnostic technologies, including X-rays, CT scans, and ultrasounds, have successively emerged, capable of clearly and accurately identifying the structural states of internal organs in the human body. However, in resource-constrained environments, where high-tech apparatuses such as ultrasound machines may be restricted or unavailable, stethoscopes remain in widespread use because of their accessibility and cost-effectiveness. Furthermore, stethoscopes offer a rapid and efficient way to collect essential physiological information about the human body in numerous clinical scenarios. Stethoscopes facilitate a swift initial evaluation of a broad spectrum of conditions, particularly those on the heart and lungs, during emergencies or routine examinations, which is crucial for immediate insight into a patient’s health status.

#### 3.1.2. Electronic Stethoscope

The application of traditional acoustic stethoscopes is sometimes constrained by the human ear’s limitations in sound recognition; for instance, frequencies below 50 Hz may be imperceptible. This restriction has prompted the advent of electronic stethoscopes, which offer enhancements over traditional stethoscopes [33]. While the detection and analysis of heart or pulmonary sounds with a traditional acoustic stethoscope significantly depend on the clinician’s experience, electronic stethoscopes convert acoustic sounds into electrical signals that can be amplified, recorded, and replayed, providing a more detailed and accessible auditory analysis [34].

Although electronic stethoscopes and traditional acoustic stethoscopes share a similar appearance, their operating principles are quite different. Electronic stethoscopes have a contact microphone in the chest piece that converts skin vibrations into an electrical signal. This signal is then amplified, filtered, and fed to a speaker or headset for optimal listening [35]. The electronic amplification technique overcomes the low sound level problems typically associated with traditional acoustic stethoscopes, and the converted electrical signals can be digitized for further processing and transmission, which is valuable for diagnostic and research purposes [36]. In terms of structure, there are three main modules in a general electronic stethoscope system, namely the data acquisition module, preprocessing module, and signal processing module. For the data acquisition module, the sound signals recorded by the electronic stethoscope are converted into digital signals and sent to the preprocessing module. The preprocessing module will normalize and segment the signal, and the signal processing module will extract and classify the features of the signal [36].

**Figure 2:**
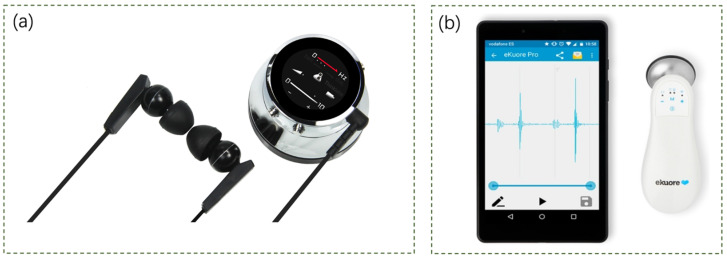
Examples of electronic stethoscopes. (**a**) The Thinklabs One [37]. (**b**) The eKuore stethoscope [37].

There is a wide range of electronic stethoscopes on the market today. Here, we have selected a few representative ones to introduce. The Thinklabs One (Thinklabs, 6500 S Quebec St., Suite 210, Centennial, CO 80111, USA) (Figure 2a) is a digital stethoscope with a wide selection of filters that can be used to capture sound from the heart, lungs, and other parts of the body. It offers the capability to listen to completely unfiltered sound along with multiple filter options, and it boasts a sound amplification capacity of up to 100 times. The volume and filter controls can be used through the device’s buttons. Users can listen to body sounds through external headphones or record from devices with on-board devices (laptops, smartphones, etc.). The eKuore stethoscope (eKuore Medical Devices, Burjassot, Valencian Community, Spain) (Figure 2b) utilizes a Wi-Fi connection to communicate with any smartphone or tablet via a free dedicated app. On these devices, cardio-voice ECGs can be recorded and viewed in real-time or shared with remote users. Additionally, headphones can be used for auscultation as the device is equipped with a 3.5 mm jack connector.

The 3M™ Littmann CORE Digital Stethoscope (3M Health Care, St. Paul, MN 55144, USA) can be switched between analog and amplified listening modes. The amplified listening mode provides up to 40 times more amplification than the analog mode, and active noise reduction reduces unwanted background sounds. It can also be connected to Eko software (Eko app, version 5.8.0) to visualize and share heart sound waveforms, allowing users to save and annotate 15, 30, 60, or 120-s recordings. The Stethee Pro Digital Stethoscope (M3DICINE, Queensland, QLD 4113, Australia) is equipped with five specially designed filters that facilitate a frequency response ranging from 20 Hz to 2 kHz. It also allows for 24× and up to 96× amplification of the sound. The Stethee Pro Digital Stethoscope captures cardiac vital sign data in less than 20 s and provides a complete analysis of systolic and diastolic heart sounds because it automatically captures and measures systolic and diastolic heart sounds in milliseconds.

### 3.2. Wearable Devices

In recent years, wearable devices have made significant strides in the field of health monitoring, particularly in personal health management and telemedicine services. Modern wearable technology, with its integration of high-precision sensors, advanced data processing capabilities, and seamless connectivity features, not only provides users with real-time health tracking but also has the potential to predict underlying health issues by analyzing physiological data [38]. The networking capabilities of these devices enable healthcare providers to monitor patients’ health remotely, refine treatment plans, and offer timely interventions when necessary. Wearable devices are progressively becoming an indispensable part of health management and medical services, greatly enhancing the accessibility and personalization of healthcare.

Wearable acoustic sensing devices introduce a new dimension to health monitoring by capturing and analyzing sounds emitted from the body. These devices can precisely monitor the sounds of the heart and respiratory system, identifying signs of arrhythmias or respiratory diseases, and also assess sleep quality, for instance, by diagnosing sleep disorders through the analysis of snoring sounds. The non-invasive nature and the continuous monitoring capability make these devices an ideal choice for home health management for the elderly and chronic disease patients while also offering healthcare providers the convenience of remote patient monitoring [39]. As technology advances, the application of wearable acoustic sensing devices in health monitoring is set to broaden, providing a wealth of data support and deeper insights into daily healthcare and professional medical diagnosis [40].

Recent breakthroughs in material science, micro–nanofabrication, and bionics have spurred the development of novel wearable acoustic sensing devices. These innovative forms, including electronic skin (e-skin), electronic fabrics (e-fabrics), and bionic devices, boast a suite of expanded functionalities and features. They are engineered to be highly conformable, lightweight, comfortable, and breathable while delivering precision in data capture. Such advancements are dramatically enhancing the integration of these wearables into everyday life, ensuring minimal intrusion and maximum benefit for users in health monitoring scenarios. Herein, we will summarize and explore some of the emerging wearable acoustic sensing devices from skin patches, fabrics, and bionic MEMS.

#### 3.2.1. Acoustic Skin Patches

Skin patch wearable sensors can be integrated with the human body in various forms (including tattoos and adhesive patches) to perform in vivo sensing, data logging, and computation using a mobile device. These patches are typically flexible and wireless, continuously collecting physiological data such as heart rate variability, body temperature, and blood pressure [41]. Acoustic sensing via skin patches represents a burgeoning field within biomedical engineering, concentrating on creating sensors that adhere to the skin and are adept at capturing and interpreting acoustic signals. Designed to conform closely to human skin, skin patch acoustic sensors boast an array of biomedical applications thanks to their flexibility, sensitivity, and non-invasive nature.

**Figure 3:**
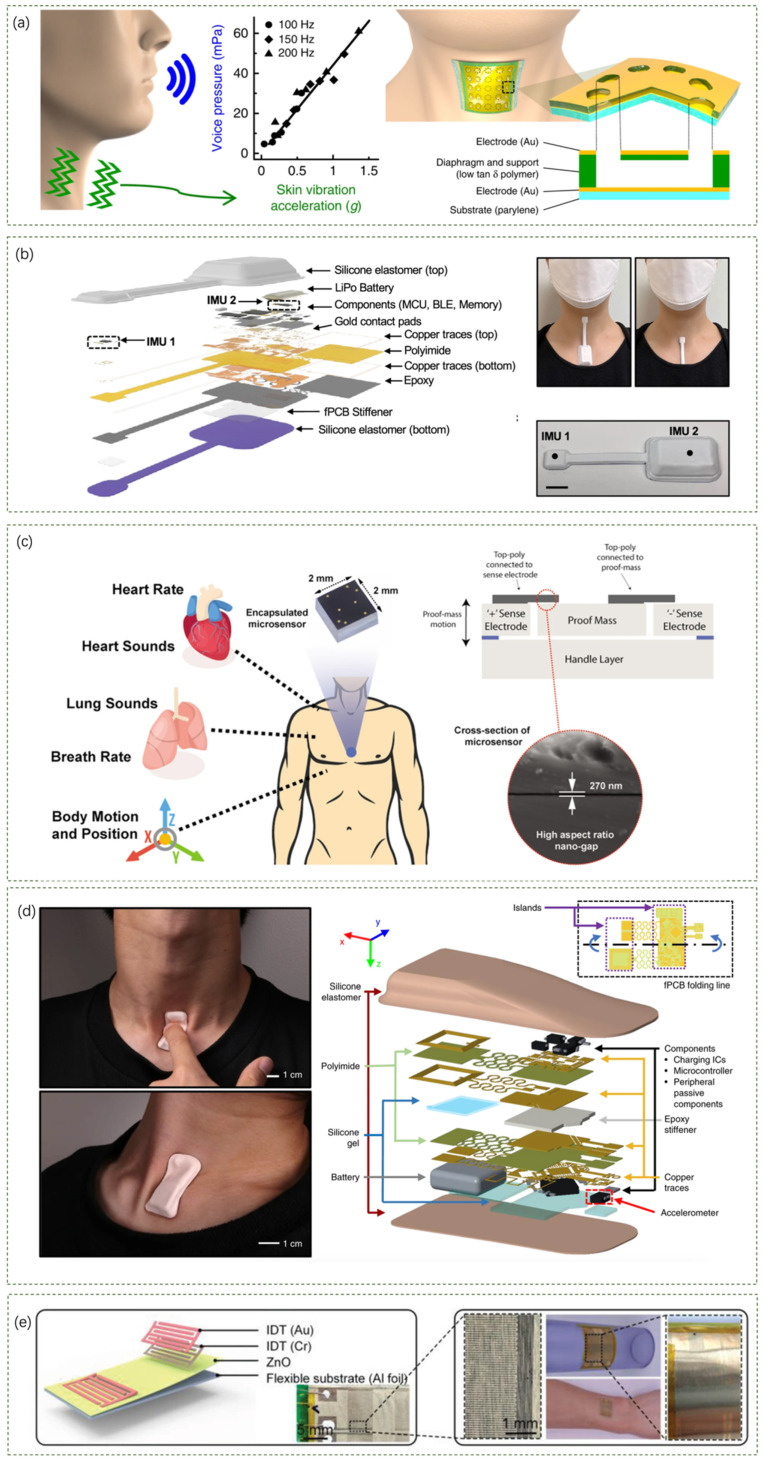
Wearable acoustic skin patch devices. (**a**) An ultrathin, comfortable, and vibration-responsive electronic skin from Siyoung Lee et al. [42]. (**b**) Soft skin-interfaced mechano-acoustic sensors from Youn J. Kang et al. [43]. (**c**) Precision wearable accelerometer contact microphones from Pranav Gupta et al. [44]. (**d**) A soft wireless device placed at the suprasternal notch from KunHyuck Lee et al. [45] (**e**) Multifunctional and Wearable Patches from Qian Zhang et al. [46].

Skin patch sensors are commonly used to detect activities on the surface of the skin by closely fitting the sensor to the skin, which can accurately recognize subtle motion signals generated by skin vibrations. An ultrathin, comfortable, and vibration-responsive electronic skin (Figure 3a) for quantitative sound recognition proposed by Siyoung Lee et al. can be used to detect skin acceleration, which is highly linearly correlated with sound pressure [42]. Their device consists of a cross-linked ultrathin polymer film and a pore-like diaphragm structure, which provides a flat frequency response, high sensitivity, and excellent skin fit, enabling accurate speech recognition on rough and curved skin surfaces. In turn, Youn J. Kang et al. have proposed a wireless wearable technology that utilizes miniature soft wireless sensors mounted near the neck to capture and monitor subtle and rough movement and vibration processes on the skin surface (Figure 3b) as a way to achieve continuous monitoring of respiratory activity and swallowing [43]. Validation studies on healthy adults and patients with dysphagia have shown that the measurements are comparable to existing clinical standard devices.

Regarding sensor selection and utilization, some teams have demonstrated innovative development and integration of sensors/sensing devices with different principles and scope of use. Using accelerometers as a prime example, Pranav Gupta et al. presented a wearable accelerometer contact microphone (ACM) for longitudinal monitoring of mechano-acoustic cardiorespiratory signals (Figure 3c) [44]. The ACM combines the properties of an accelerometer and a contact microphone to acquire broadband physiological signals related to the cardiorespiratory system. When tested on control subjects and patients with pre-existing conditions, the ACM sensor demonstrated high sensitivity, wide bandwidth, and the ability to monitor a wide range of health factors related to the cardiopulmonary system. Another study using accelerometers was presented by KunHyuck Lee et al. [45]. In their study, a wireless device with an integrated high-bandwidth triaxial accelerometer was mentioned (Figure 3d). The main electronic system of this device consists of a triaxial digital accelerometer, a microcontroller for data acquisition and wireless communication, and wireless inductive charging circuitry. Its engineering mechanics are designed to provide high data fidelity and a comfortable, non-irritating skin interface. In addition, they use frequency domain analysis and machine learning to extract quantitative physiological information from collected data, which can be used to monitor health, quantify sleep behavior, measure exercise performance, and guide rehabilitation programs.

On the use of the piezoelectric effect, Qian Zhang et al. demonstrated a wireless platform (including sensing, transmitting, and receiving) based on flexible piezoelectric acoustics (Figure 3e) for integrated sensing, localization, and underwater communication [46]. The platform is made possible by flexible piezoelectric acoustics, which utilize high-frequency (approx. 13 MHz) excitation to generate Lamb waves for respiration monitoring and low-frequency excitation (approx. 20 kHz) for communication and positioning applications. The platform enables real-time respiration monitoring, wireless communication within a range of 2.8 m at 200 bps or 4.2 m at 25 bps, and distance measurements within a range of 100 m (maximum error of 3 cm).

**Figure 4:**
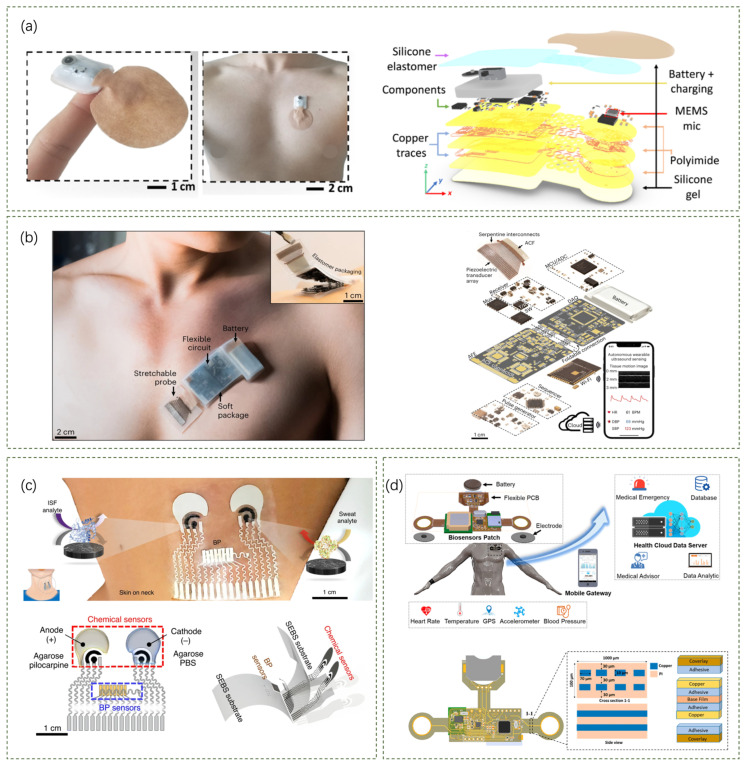
Wearable acoustic skin patch devices. (**a**) A soft wearable stethoscope from Sung Hoon Lee et al. [47]. (**b**) A fully integrated autonomous ultrasonic-system-on-patch (USoP) from Muyang Lin et al. [48]. (**c**)The stretchable integrated BP–chemical sensing patch from Juliane R. Sempionatto et al. [49]. (**d**) A wireless and flexible biosensor from Duc Tri Phan et al. [50].

There are also teams that have researched and innovated on the stethoscope in terms of skin-patch wearables. As shown in Figure 4a, Sung Hoon Lee et al. explored a soft wearable stethoscope (SWS) for continuous auscultation and automated disease diagnosis [47]. They designed a soft wearable system to provide real-time, wireless, continuous auscultation, solving the problem of digital stethoscopes that are both bulky and do not fit the skin. The SWS has excellent soft-mechanical properties, including an elastomeric enclosure with an inner silicone gel (300 μm thickness and 4 kPa Young’s modulus) and densely packed layers of soft materials and electronic components, including a MEMS microphone sensor, flexible thin-film circuits, a rechargeable battery, and a Bluetooth low-energy (BLE) unit for wireless data transmission.

In addition to the above research advances, it is worth mentioning that ultrasound is also an effective acoustic sensing technology involving the use of sound waves with frequencies higher than the audible range for humans (above 20 kHz) to detect objects and measure distances. Known for its non-invasive and safe methods, ultrasonic sensing technology is widely used in medical imaging, industrial inspections, and automotive sensors, with high resolution, depth penetration, and real-time imaging characteristics. Recently, some teams have successfully combined ultrasonic sensing technology with wearable technology to build an effective tool for medical diagnosis and health monitoring. A fully integrated autonomous wearable ultrasound patch (USoP) system that monitors deep tissue signals from moving objects is presented by Muyang Lin et al. [48]. Figure 4b shows a photograph of the encapsulated USoP mounted on the chest and a schematic of the design of the USoP, including the stretchable ultrasound probe, flexible control circuitry, and battery. The ultrasound probe consists of a piezoelectric transducer array, serpentine interconnect electrodes, and an anisotropic conductive film. The USoP is capable of continuously tracking physiological signals from tissues up to 164 mm deep and monitoring central blood pressure, heart rate, and cardiac output of a moving subject for up to 12 h.

In addition to the unique design and use of a particular sensor, a number of teams have adopted the idea of combining multiple sensors in one skin patch device. A non-invasive wearable sensor proposed by Juliane R. Sempionatto et al. consists of an ultrasonic sensor for monitoring blood pressure and heart rate and an electrochemical sensor for detecting biomarkers (Figure 4c) [49]. The sensor can simultaneously monitor blood pressure, heart rate, and various biomarkers such as glucose, lactate, caffeine, and alcohol. Another example is a wireless, flexible biosensor patch proposed by Duc Tri Phan et al. for continuous, longitudinal monitoring of different physiological signals, including body temperature, blood pressure, and electrocardiography (ECG) [50]. Their biosensor module includes multiple sensors such as a PPG sensor, 9-axis accelerometer, clinical grade temperature sensor, ECG sensor, and GPS module. It also includes a low-power microcontroller unit for collecting and processing health data. Figure 4d shows an overview of the wearable biosensor patch applied to the Internet of Medical Things (IoMT), along with the design and structure of the biosensor device. The patch design is small and lightweight (less than 0.1 mm in thickness and less than 5.0 g in weight) to ensure maximum comfort and stability when applied to the curved skin surface of the chest. The mechanical properties of the patch (e.g., stretchability and skin compatibility) are optimized to minimize irritation and discomfort during prolonged wear. In addition, they also presented an IoT-connected healthcare platform for remote monitoring and attempted to develop a deep learning architecture for non-invasive continuous blood pressure monitoring.

#### 3.2.2. Acoustic Fabrics

Fabric acoustic sensing is an innovative field that combines textiles with acoustic technology to enable fabrics to sense and interact with sound. These sensors offer unique qualities such as flexibility, breathability, abrasion resistance, and sensitivity to sound. The sound-sensitive fibers integrated with these fabrics, such as piezoelectric fibers, are the key components that are capable of converting acoustic vibrations into electrical signals. This innovative amalgamation offers a new dimension to the functionality of smart textiles, enabling them to interact with the environment in more complex and useful ways and monitor human health status in everyday life in an efficient and unobtrusive manner.

**Figure 5:**
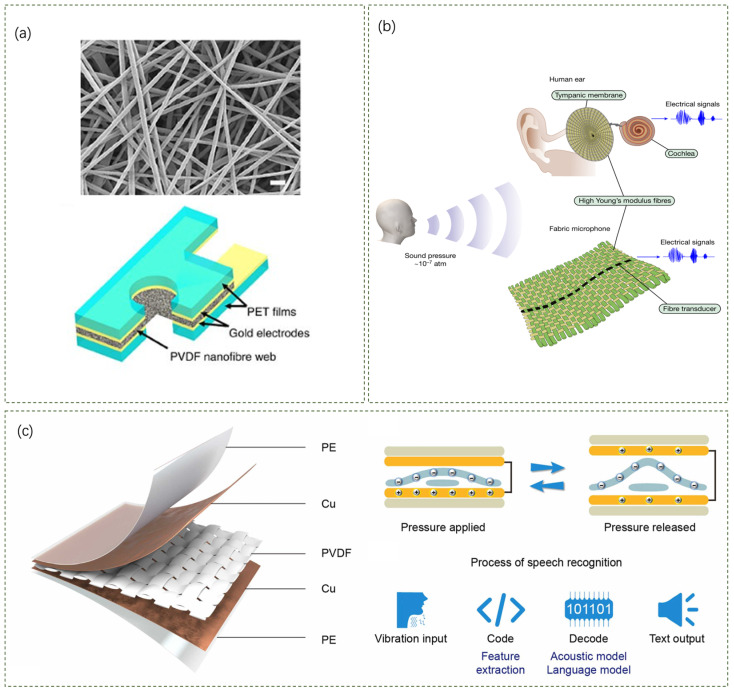
Wearable acoustic fabric devices. (**a**) High-sensitivity acoustic sensors from Chenhong Lang et al. [51]. (**b**) The fabric microphones from Wei Yan et al. [52]. (**c**) Anti-interference self-powered acoustic fabric from Jizhong Zhao et al. [54].

Piezoelectric nanofibers produced by electrospinning show remarkable electrical conversion of mechanical energy, while for acoustic–electric conversion, Chenhong Lang et al. demonstrated that electrostatically spun piezoelectric nanofiber webs have strong acoustic–electrical conversion capabilities [51]. Their study showed that sensor devices made of piezoelectric nanofibers, especially those using poly(vinylidene fluoride) as a model polymer, exhibited sensitivity up to 266 mV Pa^−1^ for low-frequency sounds. The high-sensitivity acoustic sensors are constructed by sandwiching a layer of polyvinylidene fluoride (PVDF) nanofibers between two transparent polyethylene terephthalate (PET) films, where the films are coated with metal, and the metal surface is in contact with the nanofiber layer, which serves as electrodes for collecting the electrical signals (Figure 5a). In order for the nanofibers to receive the sound waves directly, a through-hole was cut into each plastic film. This sensor structure is capable of accurately differentiating sound waves in the low to medium frequency range and has a higher sensitivity to sounds with pressure levels above 100 dB, making it ideal for noise detection.

Excellent fabric acoustic sensors can be constructed using a single piezoelectric fiber combined with an acoustic harvesting structure. Wei Yan et al. proposed a fabric that can be used as a sensitive audible microphone [52]. The fabric is woven from high Young’s modulus textile yarns and thermally stretched composite piezoelectric fibers, in which cotton warp and weft yarns and optical fibers convert mechanical vibrations into electrical signals, while the elastic cladding can improve the sensitivity. Figure 5b shows the structure and principle of tympanic membrane movement in an acoustic fabric, and the acoustic fabric is made of high-modulus Twaron and cotton yarns woven at right angles to each other, mimicking the structure of the tympanic membrane. The fabric acts as a sensitive microphone that can detect weak sound signals (e.g., human speech) down to 10^−7^ atm. Through concurrent measurements of the acoustic fiber-on-membrane, the fabric’s minimum sound detection capability of 0.002 Pa (40 dB, sound pressure level in a quiet library) is superior to that of many other thin-film-based acoustic transducers, and its sensitivity of 19.6 mV (at 94 dB and 1 kHz, which follows standards in the field) is comparable to that of existing capacitive and moving-coil microphones. The fabric measures the direction of acoustic pulses and establishes bi-directional communication between the fabrics. Due to high sensitivity to vibration and matching of skin impedance, the fabric is well suited for physiological sensing, acting as a skin-interface stethoscope, effectively capturing heart signals and providing information about the wearer’s cardiovascular system. Thus, the fabric has the potential to act as an efficient sound collector and play a new role in acoustic communication and health monitoring [52,53].

Another research has shown that fabric acoustic sensors can be constructed using multiple layers of different materials and can be realized to cope with complex external conditions. Jizhong Zhao et al. devised an anti-interference self-powered acoustic fabric (ASAF) (Figure 5c), which can be used as a wearable sound receiver in complex acoustic environments [54]. ASAF is made of polyvinylidene fluoride (PVDF), which can record human speech over a wide range of vibration frequencies and has high accuracy in speech recognition related to extreme weather conditions. Specifically, the soft-woven PVDF film as a vibration-sensitive layer enables ASAF to cover a wide frequency range of 0–5000 Hz and perform accurate recordings with 0.0045% drift and 61.1 dB signal-to-noise ratio (SNR). Meanwhile, ASAF fulfills the requirements of wearable acoustic sensors under complex acoustic environments (CAE) conditions with similarities of 92.76%, 87.69%, 88.04%, and 88.62% to the standard case, respectively. ASAF meets the technical requirements for wearable acoustic sensors, such as high sensitivity, wide response frequency, low detection limit, non-air dielectric conduction, and high mechanical flexibility, and has been proven to exhibit excellent stability and performance in various complex acoustic environments.

#### 3.2.3. Bionic MEMS Acoustic Devices

Bionic MEMS (microelectromechanical systems) acoustic sensing is a field that combines biologically inspired technology with MEMS technology to create advanced acoustic sensors. These sensors emulate the auditory mechanisms and structural characteristics found in nature, such as the ears of insects or mammals, to efficiently capture and analyze sound waves, usually exhibiting high sensitivity and broad frequency response. As MEMS devices, these sensors are remarkably compact and have very low power consumption, allowing easy integration into existing wearable devices and supporting prolonged operation times. Such features enable them to effectively capture the subtle acoustic signals emitted by the human body, assisting in disease diagnosis and health monitoring.

**Figure 6:**
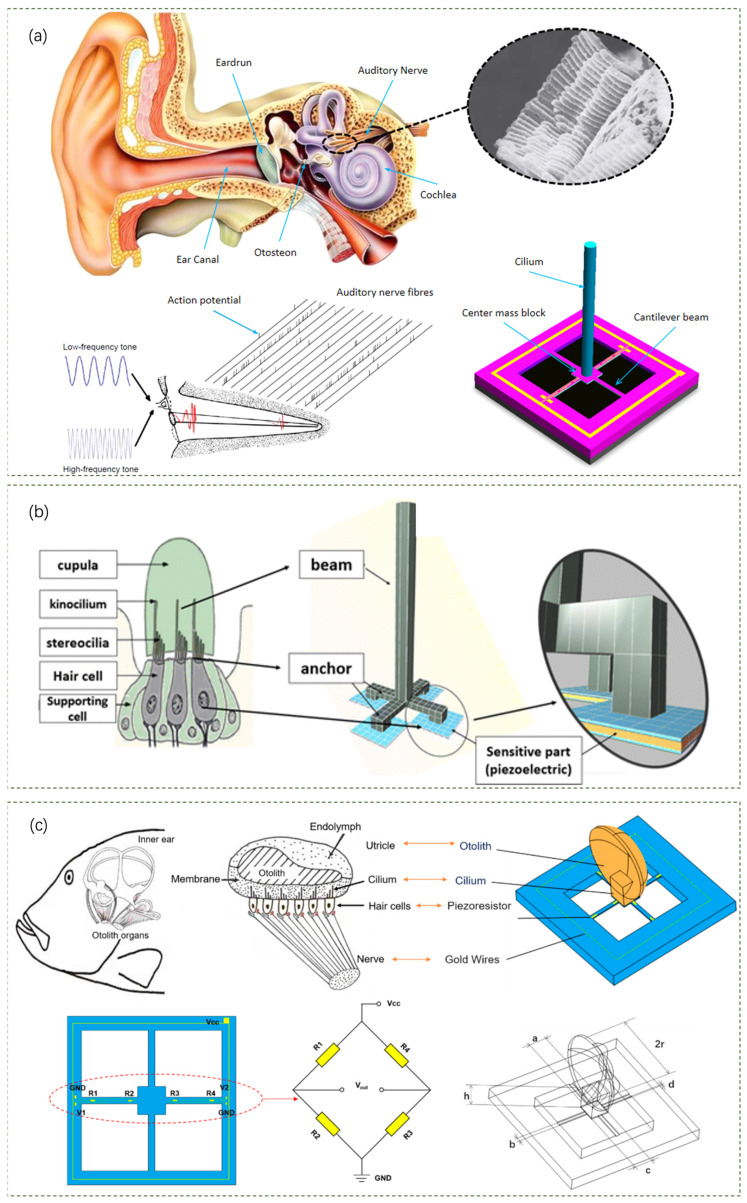
Wearable bionic MEMS acoustic devices. (**a**) A bionic structure MEMS heart sound sensor from Jiangong Cui et al. [55]. (**b**) A novel high-sensitivity MEMS piezoelectric vector hydrophone from Bahram Azizollah Ganji et al. [56]. (**c**) A novel medical acoustic sensor from Chenzheng Zhou et al. [57].

As a human hearing organ, the human ear has a subtle and efficient structure for picking up sound. In the imitation of the human ear, Jiangong Cui et al. discuss the design and optimization of a bionic MEMS heart sound sensor based on the three-dimensional ciliary bundle structure of human ear hair cells [55]. Figure 6a shows the schematic structure of the human ear, the three-dimensional ciliary bundles of hair cells, the sound-sensing mechanism of auditory nerve fibers, and the bionic MEMS sensor, respectively. The sensor design is based on the sound pickup mechanism of the human ear and utilizes MEMS technology. The height and radius of the bionic cilia are identified as key parameters affecting the sensor’s natural frequency and sensitivity. Structural optimization is performed through finite element simulation to improve sensitivity while adding a globule to the cilia is proposed to enhance sensitivity further.

Similar to the three-dimensional ciliated bundle structure of the human ear, Bahram Azizollah Ganji et al. proposed the design and modeling of a novel high-sensitivity MEMS piezoelectric vector hydrophone [56]. Figure 6b shows a schematic of the neuromast organ in the lateral line system of an aquatic vertebrate (the cilia in the neuromast help fish to perceive turbulence and sounds in the water) and the structure of the MEMS sensor. The authors used beam structures that mimic cilia and piezoelectric materials that mimic neuromast cells to detect the direction of sound in low-frequency situations. The sound pressure causes the beam structure to bend, enabling sound direction detection. This new structure not only detects the direction of the received sound but also increases the generated voltage and the sensitivity of the sensor. The experimental results show that the new MEMS piezoelectric hydrophone built by the authors’ team has improved performance with higher sensitivity (−191 dB) and applicable frequency range (10.4 kHz) and is able to detect sound waves in both directions. This bionic MEMS sensor provides important feasibility support for the reception and analysis of sound signals in liquid environments (underwater environments).

In the study of the fish ear mechanism, Chenzheng Zhou et al. found that the fish ear structure could be utilized to overcome the limitations of existing medical acoustic transducers in detecting attenuated and distorted cardiac sound signals and transmitting sound better [57]. They designed and developed a novel medical acoustic transducer based on the fish ear bionic structure (Figure 6c). The bionic acoustic transducer consists of a cantilevered beam and fish ear micromechanical structure design fabricated using MEMS technology and encapsulated in castor oil to match the acoustic impedance of the human body. When the otolith structure vibrates under sound waves, these vibrations cause the cantilever beam arms to deform, and the piezoresistive sensors on the cantilever beam arms convert this deformation into an electrical signal output. The acoustic performance test results showed that the flatness of the electret microphone and MEMS bionic acoustic sensor were 3.41 dB and 1.76 dB, respectively, and the authors’ team’s MEMS bionic sensor outperformed the electret microphone at 20–100 Hz. For the detection of heart sounds, the signal-to-noise ratios of the MEMS sensor and the 3M electronic stethoscope were 38.6 dB and 37.6 dB, respectively, with the MEMS sensor being slightly higher than the 3M electronic stethoscope by 1 dB. The experimental results show that the MEMS bionic transducer with a bandwidth of 20 Hz–2 kHz has better linearity and frequency response compared with an electret microphone, which can efficiently capture cardiac sound signals with a high signal-to-noise ratio.

## 4. Applications for Human Health Monitoring

After showcasing a variety of portable and wearable acoustic sensing devices, our attention shifted to the exploration of these sensors for monitoring human health and their ability to detect sounds from various parts of the body effectively. Human body sounds emanating from different organs exhibit a wide range of diversity. Our primary focus is on three categories of body sounds most frequently utilized in clinical environments: heart sounds, lung sounds, and gastrointestinal sounds. These sounds play a critical role in diagnostic and monitoring processes, acting as key indicators of a person’s physiological state and possible health issues. Here, we have concisely outlined the characteristics of these three sound types and presented an array of devices specifically designed to monitor such acoustic signals within the body. Table 2 summarizes the sources, characteristics, clinical applications, and detection targets of heart sounds, lung sounds, and gastrointestinal sounds. Heart sounds generated by myocardial motion, and valve activities are used to detect cardiac anomalies through their temporal and spectral characteristics [58,59]. Lung sounds, produced by airflow in the respiratory tract, help diagnose conditions such as asthma and COPD by analyzing normal and adventitious sounds such as crackles and wheezes [60,61]. Gastrointestinal sounds, resulting from intestinal motility and the movement of contents through the gastrointestinal tract, assist in identifying gastrointestinal disorders by examining their frequency and characteristics [62,63]. More specific information will be discussed in three subsections below.

### 4.1. Heart Sounds

Heart sounds (HS) are caused by myocardial motion and valve opening and closing, which are strongly influenced by myocardial hemodynamics and electrical activity [58]. In the early stages of cardiovascular disease, heart sound auscultation, as a means of initial screening for cardiovascular disease, can help differentiate between abnormal and normal heart sound signals, thus providing effective information to aid in diagnosing cardiovascular disease. Any dysfunction and anatomical defects in the heart can be reflected by the temporal, spectral, and morphological characteristics of heart sounds [59].

The segmentation and categorization of heart sound signals are currently the most commonly used methods for studying heart sound signals, and in general, the heart sound cycle is divided into four corresponding states [64]. In normal adults, the heart sound cycle mainly consists of the first heart sound (S1), systole (sys), second heart sound (S2), and diastole (dia). The S1 heart sound is produced when the mitral and tricuspid valves are closed during systole, which marks the beginning of ventricular systole, while the S2 heart sound is produced during diastolic closure of the aortic and pulmonic valves, which marks the beginning of ventricular diastole [65]. In the case of an abnormal HS, there may be several other signaling activities between S1 and S2, such as murmurs. The third heart sound (S3) is a rare additional sound caused by a sudden deceleration of blood inflow. The fourth heart sound (S4) is caused by vibrations of the valves, support structures, and ventricular walls [36].

Normal contraction and relaxation of the heart are the basis of blood circulation in the body. When there is an abnormality in the heart, it shows up in the heart sound signals, which vary for various diseases. For example, for several common heart valve diseases, heart sounds are manifested by systolic mitral regurgitation, diastolic mitral stenosis, systolic pulmonary stenosis, diastolic ventricular septal defect, systolic aortic stenosis, and diastolic aortic valve closure insufficiency, which often appear as murmurs. Experienced clinicians can make a preliminary diagnosis of the disease based on the abnormal part of the heart sound and perform some necessary tests for further diagnosis according to the patient’s needs [66].

**Figure 7:**
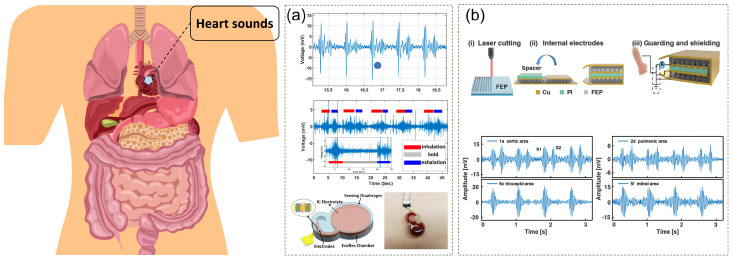
Schematic representation of heart sounds and the related wearable detection devices. (**a**) A wearable mechano-acoustic sensor for continuous cardiopulmonary monitoring from Zhiguo Zhao et al. [67]. (**b**) A flexible piezoelectric ceramic sensing system for monitoring cardiovascular and respiratory vital signs sounds from Liuyang Han et al. [68].

Zhiguo Zhao et al. present a wearable mechano-acoustic sensor (Figure 7a) for continuous cardiopulmonary monitoring [67]. This sensor is based on electrochemical redox reactions on micromachined platinum electrodes and is capable of detecting mechano-acoustic signals from the cardiopulmonary system with high sensitivity. The body of the sensor is made of Ecoflex silicone rubber material, which is stretchable, robust, and skin-compatible. The manufacturing process for the sensor included 3D printing, mixing and curing of Ecoflex, and assembly of the electrodes. The sensor’s sensitivity was measured to be about 1100 mV/g below 10 Hz, with a minimum detectable acceleration of 4.5 $\mu g\sqrt{Hz}$ at 10 Hz, which is better than most MEMS accelerometers. The sensor successfully detects heart sounds, lung sounds, and respiratory rate, making it particularly suitable for self-management of heart failure and early detection of respiratory infections such as COVID-19.

Liuyang Han et al. demonstrated a flexible piezoelectric ceramic sensing system for monitoring cardiovascular and respiratory vital signs sounds (Figure 7b) [68]. The system can detect heart, Korotkoff, and respiratory sounds in an unobtrusive manner. The fabrication process involves constructing parallel grooves on the surfaces of two FEP films, bonding the films together to form a crisscross air cavity, attaching external and internal electrodes, and folding the FEP strip with polyimide tape as the spacer layer. For heart sound detection, they successfully extracted and analyzed heart sounds related to the systolic and diastolic states of the heart and achieved simultaneous recording of heart sounds from different locations on the chest using a sensor array. They used an independent component analysis (ICA) algorithm to separate four independent components corresponding to the mitral (M1), tricuspid (T1), aortic (A2), and pulmonic (P2) valves from the mix of heart sounds to diagnose cardiac diseases accurately.

### 4.2. Lung Sounds

Lung sounds are important indicators of respiratory health and are used to differentiate between normal and abnormal breathing conditions. Listening to the lungs is a non-invasive, real-time, and informative method essential to the physical examination. Normal respiratory sounds (NRS) and adventitious respiratory sounds (ARS), such as bursts and wheezes, are essential for the diagnosis of respiratory disease. Interpretation of these sounds provides valuable information about the physiology and pathology of lung and airway obstruction.

The human respiratory cycle has two distinct phases: inhalation and exhalation. During inhalation, the diaphragm descends, the muscles contract, the volume of the chest depression increases, and high-pressure oxygenated air rapidly enters the lungs and flows to the alveoli. Expiration, conversely, is the process of releasing air from the lungs [60]. During normal respiration, air flows along the tracheobronchial tree, and the turbulent and swirling air currents in these flows produce breath sounds [61].

Abnormal lung sounds include crackles, wheezes, rhonchi, stridor, and pleural friction rubs. Crackles are short, explosive, nonmusical sounds often heard in patients with parenchymal lung diseases such as pneumonia, interstitial pulmonary fibrosis, and pulmonary edema. Wheezes are high-pitched musical sounds associated with asthma and chronic obstructive pulmonary disease (COPD), among other airway diseases. Rhonchi, on the other hand, is a low-pitched musical sound similar to snoring and usually indicates secretions in the airways [69].

**Figure 8:**
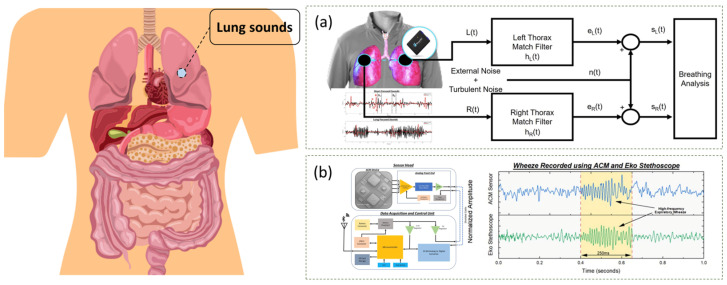
Schematic representation of lung sounds and the related wearable detection devices. (**a**) A wearable multimodal acoustic system for respiratory analysis from Lloyd E. Emokpae et al. [70]. (**b**) A wearable sensor module that uses precision accelerometer contact microphones (ACM) from Pranav Gupta et al. [71].

In detecting COPD and applying it to patients, Lloyd E. Emokpae et al. developed a wearable multimodal acoustic system for respiratory analysis (Figure 8a) [70]. The proposed wearable system utilizes a body-area sensor network with multiple sensors, including a digital stethoscope, electrocardiogram monitor, thermometer, and goniometer, which measures the signal-to-noise ratio of the acoustic spectrum as a measure of respiratory intensity. The authors present the results of a pilot study involving healthy subjects and patients with COPD that demonstrated a positive correlation between SNR values and health scale scores, and the system has the potential to enable remote monitoring and early detection of symptoms in patients with COPD.

For underlying lung lesions, abnormal lung sounds, and respiratory rates produced by multiple medical conditions, Pranav Gupta et al. demonstrated their design of a wearable sensor module that uses a precision accelerometer contact microphone (ACM) to detect pathologic mechanical sound signals in patients with lung disease(Figure 8b) [71]. The ACM sensors are designed to capture high-frequency vibrations on the skin surface caused by underlying lung lesions, and the sensor module allows intermittent and longitudinal assessment of lung sounds, breathing patterns, and respiratory rate. Through an exploratory study involving patients with exacerbations of COPD, pneumonia, and acute decompensated heart failure, the sensor module accurately captures pathologic lung sounds and provides valuable diagnostic information.

### 4.3. Gastrointestinal Sounds

Gastrointestinal sounds, also known as bowel sounds, are noises produced in the digestive tract through intestinal motility. These sounds are an important part of the physical evaluation of the abdomen and are usually examined by auscultation to determine the presence and nature of bowel sounds. The analysis of bowel sounds plays a vital role in the diagnosis of various gastrointestinal disorders.

In different diseases of the digestive organs, scholars have categorized the different bowel sounds that can be heard as splashing, rattling, or rustling, conductive murmurs of respiration, and rhythmic pulsations of the aorta [72]. By 1975, Daniel Delle et al. analyzed bowel sounds for the first time using a computer and classified bowel sounds into three types using the duration of bowel sounds as a classification index [62,73]. In 2018, Xuhao Du et al. identified them based on their duration and extended the spectrogram information based on short-time Fourier analysis to classify bowel sounds into five types. These five types of bowel sounds were categorized as a single burst, multiple bursts, continuous random sound, harmonic sound, and a combination sound [63].

Under normal conditions, bowel sounds are low and gentle and occur irregularly at 5 to 35 beats per minute. When the bowel is not functioning properly or is obstructed, the bowel sounds are usually louder or, softer, more frequent, or higher in pitch. Overactive bowel sounds appear as loud, high-pitched, rapid sounds that indicate increased bowel activity, and they may occur in conditions such as gastroenteritis or early bowel obstruction. Weakened or absent bowel sounds indicate decreased bowel activity, which may occur in conditions such as intestinal obstruction or advanced bowel obstruction [74,75].

**Figure 9:**
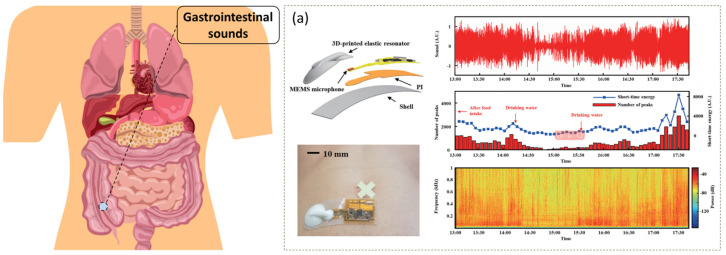
Schematic representation of bowel sounds and the related wearable detection devices. (**a**) A flexible, skin-attachable wireless acoustic device for real-time monitoring and assessment of bowel sounds from Fengle Wang et al. [76].

Fengle Wang et al. present a flexible, skin-attachable wireless acoustic device for real-time monitoring and assessment of bowel sounds (Figure 9a) [76]. The device integrates 3D-printed elastomer resonators with flexible electronics that can be attached to the abdominal surface without degrading performance during breathing. Clinical tests on normal volunteers and patients with intestinal disorders have shown that the device is able to capture the characteristics of bowel sounds. A wearable system consisting of a wearable bowel sound capture device and a bowel sound detection algorithm was proposed by Yuzhe Qiao et al. The wearable device consists of two stethoscopes and a recorder mounted on a posture correction belt. The system captures body sounds using a dual-channel stethoscope-enhanced microphone, then extracts time and frequency domain features for analysis, and finally uses a trained classifier for bowel sound detection. Test results show that the system achieved a detection accuracy of 85.7% in a real-world environment [77].

Table 3 shows several applications of human health monitoring devices mentioned above and summarizes their mechanism, application, and key performance indicators. Each device utilizes distinct mechanisms to capture physiological sounds from different body parts and translate them into valuable health metrics. For cardiopulmonary monitoring, devices such as wearable mechano-acoustic sensors detect heart and lung sounds with high sensitivity and low noise, making them suitable for continuous monitoring of cardiac and respiratory health. The dynamic sensitivity and responsiveness of the piezoelectric ceramic system allow for accurate detection of cardiovascular and respiratory vital signs, aiding in the diagnosis and monitoring of related conditions. Respiratory analysis and COPD monitoring benefit from multimodal acoustic systems that combine various sensors to capture a comprehensive array of respiratory sounds, thereby enhancing the accuracy and reliability of respiratory health assessments. Devices equipped with precision accelerometers are adept at capturing high-frequency vibrations from lung sounds, providing critical data for diagnosing lung diseases through the detection of pathological mechanical signals. For gastrointestinal health monitoring, flexible, skin-attachable acoustic devices and bowel sound capture systems are used to monitor and assess bowel sounds in real time. These devices leverage advanced materials and feature extraction algorithms to deliver high accuracy and low deviation in detecting bowel sound patterns, which are crucial for diagnosing and managing gastrointestinal disorders.

### 4.4. Sound Signal Processing for Clinical Practice

In the previous sections, we summarized some of the applications used for human health detection, where different devices and sensors are used to collect data about heart sounds, lung sounds, and gastrointestinal sounds in a clinical setting. The processing of these acoustic signals and how to effectively utilize them for clinical practice is an area of interest. Here, we discuss the methods and techniques involved in the processing and utilizing of these signals in a clinical setting, covering several key steps: preprocessing and noise reduction, feature extraction and signal analysis, clinical integration and decision support, validation, and continuous learning.

(1) Preprocessing and Noise Reduction

The first step in making acoustic signals clinically useful is preprocessing to remove noise and artifacts. This is crucial for ensuring the reliability of these data. Techniques such as adaptive filtering, wavelet denoising, and empirical mode decomposition (EMD) are commonly used. Adaptive filtering adjusts to the varying noise characteristics in real time, improving the signal-to-noise ratio [78]. Wavelet denoising is effective in separating noise from the actual signal based on their frequency differences, making it particularly useful for complex signals such as heart sounds [79]. EMD is a data-driven approach that decomposes a signal into its intrinsic mode functions, effectively isolating the noise components [80]. These preprocessing techniques are essential for ensuring that the subsequent analysis is based on clean and accurate data.

(2) Feature Extraction and Signal Analysis

Once the signals are preprocessed, the next step is to extract relevant features that can be used for clinical diagnosis. For heart sounds features such as the timing and frequency of S1 and S2, the presence of murmurs, and other abnormal sounds are critical [81]. Techniques such as the Fourier transform and Hilbert–Huang transform are employed to analyze these features in both time and frequency domains [82]. For lung sounds, identifying patterns such as crackles, wheezes, and rhonchi involves analyzing the amplitude, duration, and frequency characteristics [83]. Advanced algorithms, including machine learning models, are increasingly being used to automate this feature extraction process. These models can be trained to recognize specific patterns associated with different pathologies, thus aiding in rapid and accurate diagnosis [84].

(3) Clinical Integration and Decision Support

Integrating these processed and analyzed data into clinical practice involves creating decision support systems that can assist healthcare providers in making informed decisions. Electronic Health Record (EHR) systems can be augmented with these decision support tools to provide real-time analysis and alerts based on acoustic data. For instance, an EHR system integrated with heart sound analysis can alert a clinician to potential signs of heart failure or valve abnormalities. Similarly, systems that analyze lung sounds can provide alerts for conditions such as pneumonia or COPD exacerbations based on detected acoustic patterns [85]. These integrations help provide timely and accurate diagnoses and improve patient outcomes.

(4) Validation and Continuous Learning

For these technologies to be reliable, continuous validation and learning are crucial. This involves regular updates to the machine learning models based on new data and clinical outcomes. Techniques such as cross-validation and external validation with independent datasets are employed to ensure the robustness of the models [86]. Furthermore, feedback from clinical practice can be used to refine these models, making them more accurate and adaptable to different patient populations [87]. This continuous learning loop is essential for maintaining the efficacy and reliability of acoustic signal analysis in clinical practice.

## 5. Conclusions and Perspectives

In the field of human health monitoring, human physiological acoustic signals are important indicators of disease and health status. Compared with other traditional monitoring methods, the detection of acoustic signals is characterized by non-invasiveness, ease of use, and high efficiency. With the development of acoustic sensing technology and the continuous optimization of hardware and software, portable and wearable acoustic sensing devices are emerging and integrating with real-world medical needs, playing an important role in clinical diagnosis and health monitoring.

This review summarizes the foundational principles of acoustic sensing technologies, including capacitive, piezoelectric, and triboelectric mechanisms. It categorizes the diverse array of devices into portable and wearable acoustic sensing devices, discussing their practical applications. For portable devices, we compare traditional stethoscopes with electronic stethoscopes, focusing on their usability and portability in clinical diagnostics. Wearable devices, such as acoustic skin patches, acoustic fabrics, and Bionic MEMS acoustic devices, are highlighted for their patient comfort and suitability for prolonged monitoring. The application of these devices in human health monitoring involves capturing and analyzing acoustic signals from the heart, lungs, and gastrointestinal tract, with examples provided to illustrate their use in practice. However, these portable or wearable acoustic sensing devices for human health monitoring still face a number of challenges. Here, we summarize these challenges and propose some feasible research directions, which are described in the following sub-points:

Noise Interference: Acoustic signals for health monitoring are characterized by small amplitudes, making them highly susceptible to mechanical and electromagnetic noise. This interference can stem from friction between the device and hosting tissue or from environmental electromagnetic disturbance [40,88]. To address this, the development of sophisticated algorithms and noise-filtering techniques is crucial. These advancements can significantly enhance signal processing capabilities, thereby improving the accuracy of acoustic sensors used in health monitoring. Research into adaptive noise cancellation and machine learning-based noise reduction could provide robust solutions [58,60].

Motion Artifacts: Wearable acoustic devices often encounter motion artifacts due to mechanical stresses caused by body movements. These artifacts can displace sensor components and introduce significant noise, leading to inaccurate measurements [58,60]. Integrating advanced motion compensation algorithms and designing sensors with improved mechanical flexibility can mitigate these issues. Research into materials and designs that minimize displacement and enhance stability during movement is essential [63].

User Comfort and Acceptance: The design of wearable health monitoring devices must prioritize ergonomics to ensure user comfort and acceptance. Devices that restrict movement or cause psychological discomfort can lead to low compliance, reducing the effectiveness of continuous health monitoring [58,88]. Innovations in ergonomic design and the use of comfortable, flexible materials can alleviate these concerns. Research into biocompatible materials and wearable electronics that conform to body contours will improve user comfort and acceptance [63,76].

Data Privacy and Security: Collecting and storing acoustic health data raises significant privacy concerns. Ensuring data security through robust encryption and authentication methods is essential to protect sensitive information from breaches [63]. Implementing advanced encryption methods and robust authentication protocols will address privacy concerns and ensure compliance with regulatory standards. Research into secure data transmission and storage technologies, as well as the development of ethical and regulatory frameworks, is necessary to safeguard patient information [59].

Standardization and Durability: Current methods for calculating acoustic sensor sensitivities lack standardization, complicating performance comparisons across different devices [40,58]. Moreover, standardized methods for testing sensor durability under various sound pressures and frequencies are required to ensure their long-term reliability. Establishing universal standards for these parameters is crucial for the consistent evaluation of sensor performance. Research into developing comprehensive testing protocols and international standards will facilitate the adoption of reliable and durable acoustic health monitoring devices.

Material Limitations: The effectiveness of acoustic sensors is often constrained by the materials used. For instance, electret microphones require materials that can retain charge over extended periods to maintain stable performance [63,88]. Piezoelectric sensors depend on materials with high piezoelectric coefficients to enhance sensitivity, while triboelectric sensors require materials with significant differences in electron affinity to maximize energy conversion efficiency. Research into new materials and coatings that improve charge retention, sensitivity, and energy conversion efficiency is essential.

In addition to the above-mentioned challenges, we have investigated the market for portable and wearable acoustic devices used for human health monitoring. The demand for portable and wearable health monitoring devices is significantly driven by the rising prevalence of chronic diseases, increased public awareness of health and wellness, and the necessity for continuous health monitoring solutions. These factors have spurred interest in innovative devices capable of providing accurate, real-time health data while ensuring user comfort and ease of use. The surge in telemedicine and remote health monitoring, particularly accelerated by the COVID-19 pandemic, has underscored the critical need for reliable wearable health technologies.

Despite the high demand, there exists a considerable gap between early-stage devices (low Technology Readiness Level, TRL) and fully market-ready products. Many devices are still in the research and development phase and face challenges in achieving commercial viability. These challenges include maintaining consistent performance, ensuring user comfort, and safeguarding data security. The transition from prototype to market-ready device is often hindered by the need for rigorous standardization in sensitivity measurement and durability testing, as well as the development of advanced materials and device structures that can meet the stringent requirements of health monitoring applications.

To address these challenges and meet market demands, it is crucial to integrate advanced materials, improve data processing algorithms, and develop multifunctional systems within portable and wearable acoustic sensing devices. This integration can enhance the reliability, sensitivity, and overall performance of these devices. Furthermore, fostering collaboration between researchers, clinicians, and industry professionals is essential for developing innovative health monitoring devices that are not only technologically advanced but also user-friendly and compliant with health regulations. Such collaborative efforts can expedite the transition of innovative devices from laboratory settings to commercial markets, ultimately benefiting end-users and healthcare providers.

In summary, the acoustic sensing devices discussed in this paper incorporate diverse sensor technologies, offering new possibilities for real-time, non-invasive health monitoring, particularly in detecting and analyzing sounds from different human organs. Despite their potential, challenges such as device accuracy, user-friendliness, and data privacy remain. Future research should focus on enhancing the detection of specific physiological signals and integrating comprehensive physiological assessments. Moreover, advancements in data analysis and algorithmic processing of acoustic signals should be noted. The integration of artificial intelligence, such as machine learning and deep learning, with these devices promises to revolutionize clinical diagnosis and health monitoring by improving accuracy, intelligence, and convenience.

## Figures and Tables

**Table 1:** Comparison and summary of different acoustic sensors.

Acoustic Sensors	Principle	Characteristics	Limitations	Applicability
Condenser Microphones	Sound waves cause a diaphragm to vibrate, changing the capacitance	High sensitivity, wide frequency response, broad dynamic range, low mechanical vibration sensitivity	Requires external power (phantom power), complex manufacturing process, high-cost	High-fidelity recording (TV, broadcasting, film, theater), precise acoustic measurements
Electret Microphones	Similar to condenser microphones, but with a diaphragm of pre-polarized electret material	Compact, stable, good vibration resistance, cost-effective, no external power required	Narrower dynamic range, susceptible to environmental conditions (humidity, temperature)	Portable and remote recording applications, everyday audio devices (phones, computers, headphones)
Capacitive Accelerometers	Acceleration caused by sound pressure leads to the displacement of the mass block, changing the capacitance	High sensitivity, wide bandwidth, ability to measure static and dynamic signals	Requires external power, susceptible to shock and vibration, requires firm mounting	Machinery fault detection, building acoustics monitoring, heart sound capture, health monitoring
Piezoelectric Accelerometers	Acceleration caused by sound pressure leads to deformation in the piezoelectric material, generating electrical charge	Good high-frequency response, stable, simple structure, no external power required	Poor low-frequency response, static signals cannot be measured, requires firm mounting	Machinery fault detection, vibration analysis, shock testing, implantable hearing devices
Piezoelectric Acoustic Sensors	Sound waves cause the deformation in the piezoelectric material, generating electrical charge	High sensitivity, simple structure, anti-electromagnetic interference, no external power required	Poor low-frequency response, high output impedance, matching circuit required, high cost	Musical instrument pickups, structural health monitoring, medical diagnostics (ultrasonography)
Triboelectric Acoustic Sensors	Sound waves cause the contact and separation of materials, generating electrical charge	High sensitivity, broad frequency response, simple structure, cost-effective, no external power required	Susceptible to environmental vibration and humidity, high output impedance, matching circuit required	IoT, health monitoring, environmental surveillance, novel acoustic sensor research

**Table 2:** Summary of key information on heart, lung, and gastrointestinal sounds.

Type	Source of Sound	Characteristics of Sound Signals	Clinical Applications and Detection Targets
Heart Sounds	Generated by myocardial motion and valve activities influenced by hemodynamics and electrical activity.	Consists of the first heart sound (S1), systole, second heart sound (S2), and diastole. Abnormal sounds include murmurs, S3, and S4. Typical frequency range is 20–800 Hz.	Diagnosing cardiac diseases, detecting heart valve abnormalities (e.g., mitral regurgitation, aortic stenosis), and monitoring for heart failure.
Lung Sounds	Produced by airflow through the tracheobronchial tree and interactions with lung tissue.	Normal respiratory sounds (NRS) are low-pitched and soft, while adventitious sounds include crackles, wheezes, and rhonchi. Crackles are typically 100–200 Hz; wheezes are 400–1600 Hz.	Diagnosing respiratory conditions such as asthma, chronic obstructive pulmonary disease (COPD), pneumonia, and interstitial lung diseases.
Gastrointestinal sounds	Generated by intestinal motility and the movement of contents through the gastrointestinal tract.	Normal bowel sounds are low-pitched and gurgling, occurring 4–6 times per minute. Abnormal sounds include high-pitched or absent sounds. Typical frequency range is 100–500 Hz.	Diagnosing gastrointestinal conditions such as bowel obstructions, gastroenteritis, and monitoring for postoperative ileus.

**Table 3:** Comparison and summary of human health monitoring devices.

Device	Mechanism	Application	Performance
Wearable mechano-acoustic sensor (Zhao et al.) [67]	Based on electrochemical redox reactions on micromachined platinum electrodes	Continuous cardiopulmonary monitoring	Sensitivity: 1100 mV/g below 10 Hz, Min detectable acceleration: 4.5 μg√Hz at 10 Hz, Detects heart and lung sounds, respiratory rate
Flexible piezoelectric ceramic sensing system (Han et al.) [68]	Piezoelectric effect with FEP films and electrodes, using ICA algorithm to separate heart sound components	Monitoring cardiovascular and respiratory vital signs	Dynamic sensitivity of 591 pC/kPa in the pressure range 0–8 kPa and 290 pC/kPa in the pressure range above 8 kPa.
Wearable multimodal acoustic system (Emokpae et al.) [70]	Utilizes a body-area sensor network with multiple sensors, including digital stethoscope, ECG monitor, thermometer, goniometer	Respiratory analysis, COPD monitoring	Array gain of about 7 dB obtained by bilateral processing
Wearable sensor module with ACM (Gupta et al.) [71]	Precision accelerometer contact microphone capturing high-frequency vibrations on skin surface	Detecting pathological mechanical sound signals in lung disease patients	Ultra-low noise performance (<10 μg/√Hz) and wide operating bandwidth (>10 kHz) with sensor sensitivity of 271 mV/g, linear response to ±4 g, and cross-axis sensitivity of less than 3%.
Flexible, skin-attachable wireless acoustic device (Wang et al.) [76]	Integrates 3D-printed elastomer resonators with flexible electronics	Real-time monitoring and assessment of bowel sounds	Average 76.89% overall recognition accuracy and 3.29% standard deviation.
Wearable bowel sound capture system (Qiao et al.) [77]	Dual-channel stethoscope-enhanced microphone, time and frequency domain feature extraction, trained classifier	Bowel sound detection	Detection accuracy: 85.7% in a real-world environment, consisting of two stethoscopes and a recorder mounted on a posture correction belt
